# Perceptions of nurse-physician interactions: insights from medical students’ clinical internships

**DOI:** 10.1080/10872981.2025.2500560

**Published:** 2025-05-08

**Authors:** Anna Bovo, Mayra Veronese, Renzo Zanotti, Matteo Danielis

**Affiliations:** Laboratory of Studies and Evidence Based Nursing, Department of Cardiac, Thoracic, Vascular Sciences and Public Health, University of Padua, Padua, Italy

**Keywords:** Interprofessional education, nurse-physician interaction, medical students, qualitative research, interprofessional collaboration

## Abstract

**Introduction:**

Interprofessional Collaboration (IPC) brings together health and social care professionals to enhance patient outcomes through mutual respect, effective communication, and shared responsibility. However, while Interprofessional Education (IPE) is essential for improving communication and care quality, its implementation remains challenging. One major obstacle is the cultural and historical difference in how nurses and physicians perceive IPC, which can hinder effective collaboration.

**Purpose:**

To explore medical students’ perspectives of nurse-physician interactions.

**Methods:**

This qualitative descriptive study examined written reports from 406 second-year medical students enrolled at the University of Padua. To identify patterns in nurse-physician interactions, data were analysed using Bales’ Interaction Process Analysis categories through a deductive content analysis approach. Additionally, qualitative analysis software facilitated the coding process, with Bales’ category integrated into ATLAS® Search&Code for text analysis. Furthermore, this research was conducted and reported in accordance with the Standards for Reporting Qualitative Research guidelines.

**Results:**

A total of 438 student-reported detections were extracted from the reports and categorised into 12 of the Bales’ social interaction categories, revealing distinct roles and communication styles between nurses and physicians. Students detailed that nurses frequently provided opinions and orientations, reflecting a supportive role, while physicians were more inclined to seek orientation, indicating a collaborative approach. Moreover, the findings highlighted hierarchical dynamics, as nurses more likely to agree with others’ decisions, whereas physicians were less engaged in passive agreement. Moreover, students observed that stress and tension were more prevalent among physicians than nurses.

**Conclusion:**

The study highlights the complementary yet distinct roles of nurses and physicians in clinical settings, underscoring the importance of balanced teamwork. Addressing hierarchical dynamics and enhancing communication are crucial for improving both patient care and job satisfaction. Therefore, training programs should focus on mitigating these barriers and fostering open communication to build a more cohesive healthcare environment.

## Introduction

Interprofessional Collaboration (IPC) refers to the process through which different health and social care professionals work together to improve patients’ outcomes. This collaborative approach is defined by mutual respect, effective communication, and shared responsibility [1–3]. Perceptions of IPC are influenced by both organisational factors, such as role and workload, and individual factors (e.g., gender, age). Notably, discrepancies in these perceptions have been identified as major barriers to effective collaboration between nurses and physicians [4,5].

Healthcare professionals, including medical students, should be equipped with the skills to a collaborative workforce ready for clinical practice. Hence, IPC is imperative for Interprofessional Education (IPE) [6,7], which plays a vital role in enhancing communication and fostering interprofessional collaboration among healthcare professionals. IPC also serves to maximize staff engagement in clinical decision-making, ultimately improving patients’ quality of care, satisfaction, and safety [7–9]. To this end, the present qualitative descriptive study explores medical students’ perspectives on nurse-physician IPC using Bales’ Interaction Framework.

In the last decade, IPE has garnered increasing scientific attention [1,2,5,10–12]. This growing focus on IPE stems from the multifaceted and complex nature of patient care within the healthcare systems [1,2]. However, the implementation of IPE is not a straightforward process and presents numerous challenges. Herath et al. [11] conducted a systematic review of 65 studies, illustrating that IPE programmes vary substantially across 41 countries [11]. While the findings showed that many countries and academic institutions benefited from the introduction of collaborative teaching, learning, and practices through IPE programmes, challenges such as the length of professional education, limited hospital resources, faculty availability, teaching loads, and institutional or governmental power and leadership hinder IPE implementation [11]. IPE is grounded in the belief that all professions play equally important roles in the delivery of care. However, IPE programmes are not evenly distributed across healthcare professional’s education, often prioritising physicians as the primary ‘decision makers’ in clinical settings [10,13].

Building on this, the World Health Organization (WHO) recognises the value of a collaborative team in healthcare and has included in its research agenda the goal of sharing experiences and information to promote IPC [6]. Specifically, the WHO focuses on developing robust IPE frameworks that are integrated with local values and cultural traditions, aiming to enhance the quality and applicability of education across different healthcare settings [11]. This initiative has contributed to the integration of IPE into the national accreditation standards for health profession education institutions in various countries and regions [2].

The skills required for IPE have received significant support for improving collaborative actions and teamwork, fostering healthy relationships, and enhancing communication abilities [5,14]. Moreover, IPE emphasizes the importance of understanding and developing an attitude of respect for the competencies of other professionals [11]. A key milestone would be the development and integration of ‘shared pan professional knowledge’ [15] across healthcare professions’ education, which would promote a more comprehensive sense of integration and synergistic workload on a global scale [15,16].

However, there exists a tradition of different perceptions and interpretations of IPC among healthcare professionals, particularly between physicians and nurses [5]. For example, studies have highlighted differences between nurses and physicians in relational attitudes [15,17,18]. In their cross-sectional study on a sample of 355 nurses and 82 physicians in a non-academic acute care hospital in the south-western United States, Collette et al. [15] found that physicians and nurses viewed collaboration differently. Nurses were more likely to emphasize respect and bedside rounding to increase shared information, while physicians more frequently referred to role delineation, pointing to a clear division of established responsibilities. Perceptions of professional roles, stereotypes, and attitudes towards collaboration have been studied internationally [19–21]. For instance, Liaw et al. [22] conducted a pre-test and post-test study providing evidence that interprofessional simulation education, involving life-threatening scenarios, can change students’ stereotypical views of each other’s profession [19]. Additionally, a recent cross-sectional study investigated how medical students in both pre-clinical and clinical years perceived physician – nurse collaboration in a hospital setting in Saudi Arabia [8]. The findings revealed no significant differences in students’ attitudes toward physician – nurse collaboration based on clinical exposure, year of study, or age [8].

The Italian context is particularly sensitive to its cultural and historical backgrounds regarding interprofessional attitudes, with local literature and storytelling contributing to the perpetuation of stereotypes and traditional, profession-specific values [17,23]. In particular, Sollami et al. [17], in their research enrolling 355 medical and nursing students, found that both Italian nurses and physicians hold ambivalent stereotypes: nurses are perceived as more socially capable but less clinically competent and autonomous than physicians [17]. Furthermore, the traditional hierarchy and paternalistic approach continue to exert influence over interprofessional behaviours and attitudes in healthcare [10,17,24].

Alongside perceptions and role interpretations, expectations play a fundamental role, as different expectations can lead to varied attitudes towards interprofessional relationships and influence the IPE experience and learning [23,25]. Therefore, it is crucial to integrate education that emphasizes IPC into the curricula of medical and nursing schools. This integration will foster an understanding of the complementary roles of physicians and nurses while promoting the development of interdependent relationships between them [8,16]. Such an approach aims to improve patient care quality, enhance patient satisfaction, and increase safety [7–9,16]. Promoting IPC and teamwork requires embedding these principles throughout the curriculum and incorporating them into diverse learning activities to maximize their impact. This process should begin at the onset of professional internships, prior to clinical experiences that may influence students’ interprofessional attitudes. Given that collaboration plays a pivotal role in shaping medical students’ perspectives, it is essential to consistently emphasize its importance during clinical training in wards and other healthcare settings [26]. Moreover, students’ perceptions of IPE are crucial, as they significantly influence their engagement and attitudes toward interprofessional practice. The goal of IPE, which aims to challenge and reduce hierarchical and paternalistic stereotypes, necessitates its early integration into clinical training. This is particularly important for medical and nursing students, as it allows them to develop collaborative behaviours before adopting traditional role models.

The Bales’ Interaction Process Analysis (IPA) [27,28] is a widely used framework for evaluating group decision-making and problem-solving behaviours. This framework assesses participants’ interaction styles, identifying whether their behaviours are positive, constructive, and supportive or marked by antipathy and tension [29]. Its strength lies in systematically categorizing behaviours, balancing task-oriented and relationship-focused activities. At its core is the classification of behaviours ‘act by act’ [29].

IPA has been applied in previous studies to analyse teamwork and interactions in healthcare contexts [30,31]. However, in other medical settings, the Bales’ IPA framework has been criticized and replaced by alternative models. For instance, Campbell et al. [30] conducted a videotaped observational study of 412 consultations in 60 U.S. sites to develop a framework for clinician communication styles, specifically focusing on physicians and nurse practitioners [30]. They rejected Bales’ IPA, arguing that it was too specific for general clinician activities, such as history-taking or teaching. The study found minimal differences between physicians and nurse practitioners, except that nurse practitioners showed significantly more concern with psycho-social issues [30]. In contrast, we argue that Bales’ theory remains valuable for explaining healthcare interactions, as it continues to serves as a model for observing and understanding social behaviour [31]. Moreover, it provides a nuanced understanding of how nurses and physicians engage in their roles, distinguishing between socio-emotional and task-oriented behaviours. This distinction is crucial for capturing the complexity of nurse-physician interactions, which is essential for fostering interprofessional understanding for educational purposes [27,28].

A recent study [32] demonstrated the utility of Bales’ framework in analysing interaction patterns in surgical settings at micro level, showing how groups transition from task-focused behaviours to those addressing the socio-emotional needs of the group [32].

To the best of our knowledge, this is the first qualitative study focusing on nurse-physician interactions from the perspective of medical students during their initial medical internship. The study aims to provide insights into medical students’ perception of nurse-physician interactions during their early clinical internship.

The research question was: How do medical students perceive nurse-physician interactions during their first clinical internship?

## Methods

### Study design

This study adopted a qualitative descriptive design, focusing on the experiences reported by medical students. The study is reported here in accordance with the Standards for Reporting Qualitative Research (SRQR) [33].

### Participant information and setting

The study population consisted of 406 second-year medical students enrolled at the University of Padua, aged 20 to 23 years (Male = 180; Female = 226), with a predominance of females [55.7%]. As part of their initial clinical experience, the students completed an internship titled ‘Interactions with Healthcare Professions’, during which they were required to submit reports documenting their observations [23]. These observations were drawn from various clinical contexts, including medical (40.9%, *N* = 168), surgical (16.7%, *N* = 69), critical care (12.8%, *N* = 53), orthopaedic (12.8%, *N* = 53), neurological (5.9%, *N* = 24), oncological (4.7%, *N* = 19), paediatric (3.7%, *N* = 16), and other services (2.5%, *N* = 10).

### Ethical considerations

The School of Medicine at the University of Padua approved the study. The data consisted of anonymized written reports of student performance retrieved from the University archives. All students had signed a consent form upon enrolment, granting permission for their data to be used for research purposes only. The study also adhered to the principles outlined in the Declaration of Helsinki.

### Sampling and data collection

The sample consisted of written reports from second-year medical students, aimed at assessing the learning and working environment during their first clinical internship. These reports focused on the students’ experiences and interactions within that setting. Data collection for this study occurred over four academic years: 2017–2018, 2018–2019, 2019–2020, and 2021–2022. A total of 1,344 reports, each consisting of four to six pages in PDF format, were screened for analysis.

The reports contained detailed observations (detections) of various interactions, including nurse-physician behaviours. An observational grid assisted students to identify key behaviours and detect these interactions (see Table 1). Only reports that included elements of nurse-physician interactions were used in the study. Of the total reports, 406 were deemed valid for in-depth analysis, as they specifically detailed nurse-physician interactional behaviours.

**Table 1.** Student guide to detect key behaviours within the interactions.

Observe if	
The environment is considered adequate to the needs perceived by health professionals	
Communication is effective between health professionals and between health professionals and patients^a^	
There is a felt freedom to express opinions	
All health professionals in the team are aware of each other’s competencies	
Health professionals know and respect each other’s competencies	
All health professionals work independently within the scope of their responsibilities	
Relationships between health professionals are based on mutually shared modalities	
All health professionals have the space and opportunity to participate in communication	
There is a multi-professional discussion space for assistance cases in operating units^b^	

^a^Communication without interruption, respecting privacy, with neither party dominating the other.

^b^With a greater number of health professionals, such as physician, nurse, physiotherapist, psychologist, and others.

Each detection was classified into a category and assigned to the corresponding Bales’ IPA categories, as outlined in Table 2 [27,28]. Bales’ theory delineates two primary macro-areas of social interaction. The first, socio-emotional, revolves around the interpersonal dynamics among group members, which can be either positive (A) or negative (D). The second macro-area, task-oriented, involves attempted answers (B) and questions (C), concentrating on goal achievement and remaining neutral (Table 2). The four macro-areas are further subdivided into 12 categories, based on individuals’ relational behaviours: six categories for social-emotional areas, comprising three positive and three negative types of expression of influence and sociability, and six categories for task-oriented areas, focusing on question-and-answer interactions aimed at task completion. Bales’ theory also incorporates the possibility of linking together six ‘polarised interlocking functional problems’ [27,28] that apply to any interaction system. These problems are used to depict interactional challenges within the group and its problem-solving abilities. In this study, these functional problems were analysed to identify similarities and differences in the types of Bales’ categories observed across the two healthcare professions. The functional problems include: (a) orientation, (b) evaluation, (c) control, (d) decision, (e) tension management, and (f) integration [27,28].

**Table 2.** Areas and categories of Bales’ framework.

Functional area	Category: description
[A] Socio-emotional area: Positive reactions [1–3]	1. Shows solidarity: any act that shows positive feelings, gives help, reward
	2. Shows tension release: any act that reduces anxiety, shows satisfaction and jokes
	3. Agrees: any act that shows passive acceptance, understands, and concurs
[B] Task area: Attempted answers [4–6]	4. Gives suggestions: any act that offers direction, action, and autonomy for other
	5. Gives opinions: any act that advances an evaluation, analysis, expressed feeling
	6. Gives orientation: any act that offers information, clarifies, and confirms
[C] Task area: Questions [7–9]	7. Asks for orientation: any act that requests information, clarifies, and confirms
	8. Asks for opinions: any act that requires an evaluation, analysis, and expressed feeling
	9. Asks for suggestions: any act that requests direction, action for how to engage the task
[D] Socio-emotional area: Negative reactions [10–12]	10. Disagrees: any act that shows passive rejection, formality
	11. Shows tension: any act that shows anxiety, asking for help, withdraws out of field
	12. Shows antagonism: any act that shows negative feelings, defends or asserts self

### Data analysis

All 406 written reports were uploaded to ATLAS® version 9 for qualitative analysis. To identify, analyse, and report the patterns in the transcripts, two authors [AB and MV] employed the categories derived from Bales’ theory, thereby implementing a content analysis with deductive approach [34]. In IPA, the researcher’s role is to determine the frequency with which actions from each category occur during the interaction being analysed [31]. Both AB and MV are PhD candidates trained in qualitative methods, with backgrounds as registered nurses. ATLAS® Search&Code was used to apply Bales’ categories for text analysis, and an independent double-check was performed to assess the reliability of the process.

The table showing the distribution of the identified Bales’ categories was generated using the ATLAS® Code-Document Table, which allowed for a cross-tabulated combination of codes across the entire dataset. As a result, nurse-physician interactions, as perceived by students, were further classified in terms of frequencies and percentages. Quotations that were double-coded were carefully reviewed and adjusted if deemed ambiguous. Inter-coder reliability checks were performed, yielding a Cohen’s Kappa [κ] value of 0.63, indicating satisfactory agreement [35].

The data synthesis is summarized in Table 3, which includes examples of detections by medical students for each healthcare profession – physicians and nurses – extracted for each category within Bales’ framework. This encompasses a total of 12 categories across five functional areas.

**Table 3.** Data synthesis of medical students’ detections applied to Bales’ categories.

Functional area	Category	Healthcare profession	Examples of medical students’ detections
[A] Socio-emotional area: positive reactions	(1) Shows solidarity	Physicians	*‘ … and it was pleasant to notice how there was active communication between two components, the doctor taking into consideration the opinions and suggestions of the nurses or consulting them on various patient-related questions, while the nurses were responsive to the medical staff’s requests.’*
		Nurses	*‘I perceived the role of the nurse in a limited and filtered way […] I was able to appreciate their great professionalism, the strong relationship of collaboration, respect, and even friendship they establish with the doctors, and the great humanity and skill they demonstrate towards the patients.’*
	(2) Shows tension release	Physicians	*‘The physician was extremely calm, cordial, and collaborative. All the staff enjoyed interacting with him, asking questions, and even making some jokes. Everyone felt at ease and worked calmly despite the complexity of the procedure.’*
		Nurses	*‘Only one nurse seemed particularly cheerful and distracted all the staff from their crucial roles by chatting about vacations and trivial matters. While this might seem disruptive, I believe she was indispensable: the doctors were too serious and focused, and she helped to relieve some of the tension.’*
	(3) Agrees	Physicians	*‘She [the young doctor] under the direction of the anaesthesiologist, administers intravenous medications to the patient, before, during, and after the surgical procedure, to anaesthetize the patient or relieve pain.’*
		Nurses	*‘The doctor is treated with respect by the nurses, who support his decisions but remain free to give their opinion’*
[B] Task area: attempted answers	(1) Gives suggestions	Physicians	*‘The doctor, in turn, is aware that the nurse implements the instructions he has given.’*
		Nurses	*‘Furthermore, the nursing staff also cooperates in carrying out procedures promptly, even before the doctor may request it, and provides opinions and suggestions to the doctors, always with respect, maintaining their own role.’*
	(2) Gives opinions	Physicians	*‘The doctor evaluates the patient’s condition day by day, noting changes for better or worse in the patient’s health, and acts promptly on the administered therapy, if necessary.’*
		Nurses	*‘I have been able to appreciate how the nurses always communicate clearly with the doctor, expressing any concerns regarding the dosage of a medication, the required treatment, or the absence of prescriptions for some necessary tests for the patient.’*
	(3) Gives orientation	Physicians	*‘In addition to the written prescription, typically the doctor informs the nurse of any changes in the therapy and of the tests the patient was supposed to undergo that day.’*
		Nurses	*‘ … nurses update the doctors on the patients’ conditions at various times throughout the day and alert them to any alteration of parameters [e.g., elevated temperature, high/low blood pressure, altered heart rate, or saturation] and modify the therapeutic treatment as instructed by the doctors.’*
[C] Task area: questions	(1) Asks for orientation	Physicians	*‘Indeed, the doctors themselves ask the staff for feedback on the outcomes of the therapy and for some advice on how to proceed with the patient.’*
		Nurses	*‘Finally, issues of written communication between doctors and nurses were observed; it would happen that doctors provided incomplete instructions to nurses through the patients’ clinical diaries, so the nurses themselves had to then contact the doctors for more detailed explanations.’*
	(2) Asks for opinions	Physicians	*‘It is not uncommon for the doctor to ask the nurse’s opinion during the visit and for the case to be discussed together in order to reach the right conclusion and then to formulate an appropriate therapy.’*
		Nurses	*‘Several times, the nurses, noticing some shortcomings on the part of the doctor [especially regarding the daily therapy], before taking action to correct it, made sure to ask for consultation and consent from the doctor, even in writing.’*
	(3) Asks for suggestions	Physicians	[none reported]
		Nurses	*‘The nurse can provide the doctor, thanks to their expertise, with precise instructions regarding the therapy to be administered to each patient.’*
[D] Socio-emotional area: negative reactions	(1) Disagrees	Physicians	*‘…he [doctor] may be hesitant to listen to the advice of more experienced nurses who only want to prevent decisions that could be detrimental to the patient and not to undermine the doctor’s authority’*
		Nurses	*‘Sometimes nurses work reluctantly because they disagree with what has been established by the doctors and have not had the opportunity to make it clear or to be listened to.’*
	(2) Shows tension	Physicians	*‘This coldness and detachment that I have both seen during internship and experienced first-hand has challenged me as a future doctor as I have observed that patients, being in front of an operator who does not seem to be interested in the human part but only in that of a sanitary nature, I forgive trust only to the doctor’*
		Nurses	*‘Some nurses have reported not feeling entirely comfortable with certain doctors in the ward, as they seem to have too distant attitudes and do not appear to pay due attention to the indications and advice provided by the nurses themselves.’*
	(3) Shows antagonism	Physicians	*‘A sudden change in approach between the two surgeons was clearly noticed, no longer of the same opinion on how to proceed, and especially, with the operating room nurses: requests were made with much more insistence and in a rude manner.’*
		Nurses	*‘The relationship between doctors and nurses can sometimes be characterized by conflicts that can affect the efficiency of patient care.’*

### Rigour and transparency

Methodological rigour [36] was ensured through several strategies: (a) *credibility* was established by examining all available reports and involving researchers with adequate knowledge and research skills (see authors); (b) *dependability* was achieved through the use of homogeneous written questions and a clearly defined and shared internship objectives (the students’ mandate was explained and well understood); (c) *confirmability* was maintained by providing a detailed description of the data source and utilizing a reference framework for analysis; and (d) *transferability* was ensured by drawing from reports over several years, guaranteeing broad representativeness.

## Results

A total of 438 significant detections were extracted from 406 reports and encoded into Bales’ 12 categories (see Table 4). In the first area of positive reactions (A), medical students perceived nurses as more likely to agree (*N* = 77; 17.6%), while physicians were more inclined to show solidarity (*N* = 34; 7.8%), contributing to a supportive environment. In the second area, the attempted answers (B), students observed that both nurses and physicians provided orientation (*N* = 40; 9.2% and *N* = 32; 7.3%). Nurses were more inclined to offer opinions and suggestions (*N* = 40; 9.2% and *N* = 13; 2.9%), whereas physicians exhibited these behaviours less frequently. In the third area of questions (C), students noted that physicians requested orientation more than nurses (*N* = 30; 6.9% and *N* = 15; 3.4%). Finally, in the negative reaction area (D), students observed that physicians showed higher levels of tension (*N* = 37; 8.5%) and antagonism (*N* = 23; 5.2%) compared to nurses.

**Table 4.** Students’ detections of the nurse-physician behaviours based on the bales’ framework [*N* = 438].

	Physicians N = 180 [41.1%]	Nurses N = 258 [58.9%]
[A] Socio-emotional area: Positive reactions	37 [8.4]	97 [22.1]
1. Shows solidarity	34 [7.8]	16 [3.6]
2. Shows tension release	2 [0.4]	4 [0.9]
3. Agrees	1 [0.2]	77 [17.6]
[B] Task area: Attempted answers	42 [9.6]	93 [21.3]
4. Gives suggestions	1 [0.2]	13 [2.9]
5. Gives opinion	9 [2.1]	40 [9.2]
6. Gives orientation	32 [7.3]	40 [9.2]
[C] Task area: Questions	38 [8.7]	23 [5.2]
7. Asks for orientation	30 [6.9]	15 [3.4]
8. Asks for opinion	8 [1.8]	7 [1.6]
9. Asks for suggestion	0 [-]	1 [0.2]
[D] Socio-emotional area: Negative reactions	63 [14.4]	45 [10.3]
10. Disagrees	3 [0.7]	11 [2.5]
11. Shows tension	37 [8.5]	25 [5.7]
12. Shows antagonism	23 [5.2]	9 [2.1]

Table 5 highlights the functional problems identified in students’ detections of nurse-physician behaviours, emphasising how these two groups interact within Bales’ framework. In the first problem, which concerns the need for accurate information and understanding (=*orientation*), students detected that nurses frequently offered information and guidance (9.1%), but were less likely to seek information and confirmation (3.4%). Physicians showed a similar pattern, with a lower inclination to seek orientation (6.8%) compared to providing it (7.3%). In the *evaluation* problem, students noted that nurses were more likely to share their assessments and express feelings rather than ask others for input (9.1% vs. 1.6%). Physicians, however, engaged in both giving and soliciting evaluations and analyses (2.0% vs. 1.8%), showing a more balanced approach. The *control* problem, which involves influencing the direction and decisions of a collective effort, often by asserting authority or leadership, revealed that nurses were more likely to provide guidance or direction than to seek it from others (2.9% vs. 0.2%). Physicians, on the other hand, engaged in these behaviours less often, with percentages close to zero. In the *decision* problem, students perceived nurses to be more inclined to passively accept or agree with statements or actions made by others, rather than display rejection or formality (17.5% vs. 2.5%). Conversely, these behaviours were observed less frequently among physicians. This could be attributed to the differences in hierarchical structures, communication styles, and professional expectations between nursing and medical professions. With regard to *tension management*, both nurses and physicians were observed to exhibit tension more frequently than releasing it. However, physicians had a higher incidence of tension [8.4%] compared to nurses (5.7%). Lastly, in the *group interaction* problem, both nurses and physicians were observed to exhibit more solidarity than antagonism. Students noted a higher rate of solidarity among physicians (7.7%) compared to nurses (3.6%), suggesting a tendency toward positive group integration. Nevertheless, a higher rate of antagonism was observed among physicians (5.2% vs. 2.0% for nurses), indicating a potential area for addressing negative interactions within healthcare teams.

**Table 5.** Interlocking functional problems detected by medical students in nurse-physician behaviours according to bales’ framework.

Bales’ categories	Functional problems	Reported nurses’ behaviour N = 258 [58.9%]	Reported physicians’ behaviour N = 180 [41.1%]	Explanation
Gives orientation [6] vs asks for orientation [7]	Problems of orientation *Activities which indicate a need for factual orientation*	9.1% vs 3.4%	7.3% vs 6.8%	Nurses offer information and guidance to others, with a lesser tendency to seek information and confirms. Physicians balance providing information or guidance with seeking input or clarification, although they tend to favour providing information slightly more.
Gives opinion [5] vs asks for opinion [8]	Problems of evaluation *Activities which helps assessing some sort of event*	9.1% vs 1.6%	2.0% vs 1.8%	Nurses primarily engage in giving evaluations, analyses, or expressing feelings, with a lesser tendency to solicit them from others. Physicians demonstrate a modest inclination both to provide evaluations or analyses, and to solicit such input from others.
Gives suggestions [4] vs asks for suggestions [9]	Problems of control *The direction of group-action and taking a stand*	2.9% vs 0.2%	0.2% vs 0.0%	Nurses exhibit a greater emphasis on providing guidance or direction rather than seeking it from others. These behaviours are infrequent among physicians.
Agrees [3] vs disagrees [10]	Problems of decision *Decision-making activities*	17.5% vs 2.5%	0.2% vs 0.6%	Nurses have a greater tendency to passively accept or agree with others’ statements or actions, rather than showing rejection or formality. These behaviours are notably less common among physicians.
Shows tension release [2] vs shows tension [11]	Problems of tension management *Activities with some level of conflict or tension*	0.9% vs 5.7%	0.4% vs 8.4%	In both groups, there is a higher tendency to display anxiety, request help, or withdraw from the field compared to behaviours associated with tension release.
Shows solidarity [1] vs shows antagonism [12]	Problems of group integration *Activities which relate to team integration*	3.6% vs 2.0%	7.7% vs 5.2%	Both nurses and physicians exhibit behaviours indicative of solidarity, although physicians demonstrate a slightly higher frequency of such behaviours than nurses. Additionally, while both groups display behaviours associated with antagonism, physicians exhibit a slightly higher prevalence of these behaviours compared to nurses.

## Discussion

The study provides valuable insights into medical students’ observations of nurse-physician interactions during their initial clinical internship. By employing Bales’ theory of social interactions as a coding framework, the study systematically categorized and analysed these interactions. The higher frequency of students’ detections of positive socio-emotional interactions among nurses underscores their role in fostering a supportive environment, an essential factor for effective teamwork and patient care. Conversely, students reported a greater prevalence of negative socio-emotional reactions among physicians. A recent qualitative investigation showed that unprofessional and unsafe interactions between physicians and nurses are the causes of mistrust and conflict, leading to increased work pressure and burnout [37]. Similarly, the tendency observed by medical students towards negative reactions, coupled with the higher incidence of tension-related behaviours among physicians compared to nurses, may undermine team cohesion and contribute to a more stressful work environment. This finding suggests that physicians may experience heightened stress, potentially due to the demands of their role and the responsibility for critical decision-making. Addressing stress management is crucial for improving overall well-being and performance among healthcare professionals, reducing burnout, mitigating tension, improving overall team dynamics, and enhancing patient care [38].

The students’ insights revealed a higher prevalence of negative reactions among physicians during interactions with nurses. This trend may reflect a lack of appreciation for nurses’ roles by physicians, as well as disrespectful and repressive behaviours, reinforced by an authoritarian healthcare management structure that fosters unsafe interactions. A recent study suggests that, in the absence of specific training programs, the sociocultural environment significantly impacts the development of individuals’ collaboration skills [39]. Furthermore, this impact can be amplified in educational settings where medical training primarily emphasizes technical and clinical competencies over communication and teamwork skills [39,40]. From an educational perspective, it is essential to understand how medical students develop their attitudes towards interprofessional collaboration with nurses, as these are shaped by their exposure to hierarchical structures [41]. In such environments, physicians typically hold autonomy over patient care decisions. Therefore, integrating or strengthening IPE within medical and nursing curricula could enhance the understanding of the complementary roles of physicians and nurses. This approach could promote the development of interdependent relationships between physicians and nurses [8].

Based on medical students’ observations, nurses were often seen working in collaborative environments that emphasized teamwork and consensus-building. In contrast, physicians were perceived as operating with greater autonomy and decision-making authority, which led to fewer instances of passive agreements and more formal interactions. These observations highlight the hierarchical structure and professional dynamics that medical students perceive within healthcare teams. The hierarchical role students attribute to physicians’ positions in Italian healthcare could be another influencing factor contributing to the subordinate relationship between nurses and physicians [17], with nursing tasks often perceived as mechanical and technical responses to medical decision. IPE education could thus benefit from programmes that foster awareness of professional interactions from the earliest stages of training, allowing space for group dynamics and contributing to the development of high-quality IPC. Moreover, teamwork can positively or negatively impact both the quality and safety of care delivery [42]. Hierarchy between physicians and nurses can inhibit assertive communication, which is necessary for effectively addressing errors, such as violations of evidence-based treatment protocols. Healthcare teams, much like other teams operating in high-risk and dynamic environments, must encourage open communication, even when psychological safety may be limited and hierarchical norms are strong [43].

Students’ observations revealed that nurses frequently provide opinions and guidance, whereas physicians also offer guidance, though to a lesser extent, and are more likely to seek it. These differing interaction patterns, as observed by medical students, highlight the distinct perceived roles and communication styles within the healthcare team. The students’ detections of nurses’ frequent provision of opinions and orientation underscore their active role in guiding patient care and supporting team members. In contrast, while physicians also seek guidance, they tend to focus more on providing direction, reflecting a collaborative approach that emphasizes mutual understanding and information sharing. This dynamic suggests that nurses often take on a mentorship and supportive role, while physicians prioritise obtaining comprehensive information to inform their clinical decisions.

A notable finding in the present study concerns decision-making approaches: nurses were more frequently observed to accept or agree with statements or actions made by others rather than assert their own decisions. Conversely, physicians exhibited lower levels of passive agreement, suggesting differences in hierarchical influence and decision-making authority. Addressing this discrepancy is crucial for fostering an environment where collaborative decision-making is valued and all team members feel empowered to contribute actively to patient care decisions. Implementing interventions that promote shared decision-making and mitigate hierarchical barriers could enhance the effectiveness of healthcare teams.

The findings from this study could inform the design of IPE programmes that engage two or more healthcare professionals in a collaborative learning environment. Such programs could help improve health outcomes by providing insights into interaction processes and developing strategies to better prepare students for careers in the healthcare sector, where teamwork, collaboration, reflection on group dynamics, and stress management are essential competencies.

### Limitations

A limitation of the study is the limited background information available about the students, which precluded sub-analyses or the identification of potential relationships. For instance, it would have been valuable to assess whether older students demonstrated greater observational acuity compared to younger ones. Additionally, factors such as prior work experience or familial connections in clinical settings may have influenced students’ perceptions and, consequently, their observations. Moreover, the analysis was not segmented by clinical area, which could have affected the results, as interaction patterns may vary across different healthcare settings. Furthermore, the exclusion of data from the 2020–2021 academic year due to the COVID-19 pandemic, which necessitated a reorganisation of clinical rotations, may have impacted the generalizability of our findings. Expanding this research to include nursing and other health professions could provide more comprehensive understanding of interprofessional interactions. Finally, this study applied Bales’ theory as a framework for interpreting social dynamics, acknowledging that alternative models may offer different perspectives. As an example, previous studies that utilized the Jefferson Scale of Attitudes toward Physician – Nurse Collaboration (JSAPNC) to assess collaboration between physicians and nurses found no significant differences in medical students’ attitudes toward physician – nurse collaboration [8,44].

## Conclusion

This study sheds light on medical students’ perception of nurse-physician interactions during their initial clinical internships. Utilizing Bales’ theory, the findings indicate that students observed nurses frequently providing opinions and orientation, whereas physicians were more inclined to seek orientation. These detections reflect distinct yet complementary roles within the healthcare team. Nurses’ guiding and supportive behaviour contrasts with physicians’ collaborative approach to information gathering, highlighting the importance of balanced teamwork. Addressing hierarchical dynamics, decision-making processes, and stress management could improve these interactions. Based on these insights, strengthening interprofessional communication training and reducing hierarchical barriers, are crucial for improving patient care and job satisfaction. Furthermore, the present study identified areas for improvement, particularly in tension management and mitigating negative behaviours such as antagonism.

From a practical standpoint, fostering open communication is essential for creating an environment where all team members feel empowered to actively participate in patient care decisions, regardless of hierarchical structures. Regular feedback, team-building activities, and routine interdisciplinary meetings can enhance collaboration, promote mutual understanding of each profession’s roles and responsibilities, and help reduce tensions or negative interactions within the healthcare team. Additionally, the development of tailored stress management programs and strategies for healthcare professionals is crucial to addressing the high levels of stress and negative reactions frequently observed in clinical settings.

## Supplementary Material

Supplemental Material

## Data Availability

The dataset generated and analysed during the current study is available [in Italian] from the corresponding author upon reasonable request.

## References

[1] Reeves S, Pelone F, Harrison R, et al. Interprofessional collaboration to improve professional practice and healthcare outcomes. Cochrane Database Of Systematic Reviews. 2017;2018(8):CD000072. doi: 10.1002/14651858.CD000072.pub3 PMC6481564 28639262

[2] Khalili H, Thistlethwaite J, El-Awaisi A, et al. Guidance on global interprofessional education and collaborative practice research: discussion paper. A Joint Publication by Interprofessional Research Global & Interprofessional Global. Available from: https://interprofessional.global/wp-content/uploads/2019/10/Guidance-on-Global-Interprofessional-Education-and-Collaborative-Practice-Research_Discussion-Paper_FINAL-WEB.pdf

[3] Geese F, Schmitt KU. Interprofessional collaboration in complex patient care transition: a qualitative multi-perspective analysis. Healthcare (Basel). 2023;11(3):359. doi: 10.3390/healthcare11030359 36766934 PMC9914692

[4] El-Awaisi A, Yakti OH, Elboshra AM, et al. Facilitators and barriers to interprofessional collaboration among health professionals in primary healthcare centers in Qatar: a qualitative exploration using the “Gears” model. BMC Prim Care. 2024;25(1):316. doi: 10.1186/s12875-024-02537-8 39192182 PMC11348528

[5] Bowles D, McIntosh G, Hemrajani R, et al. Nurse-physician collaboration in an academic medical centre: the influence of organisational and individual factors. J Interprof Care. 2016;30(5):655–10. doi: 10.1080/13561820.2016.1201464 27388560

[6] WHO. Organization. WH. Framework for action on interprofessional education collaborative practice. Available from: https://iris.who.int/bitstream/handle/10665/70185/WHO_HRH_HPN_10.3_eng.pdf?sequence=1.2010

[7] van der Gulden R, Haan NDS, Greijn CM, et al. Interprofessional education and collaboration between general practitioner trainees and practice nurses in providing chronic care; a qualitative study. BMC Med Educ. 2020;20(1):290. doi: 10.1186/s12909-020-02206-1 32883272 PMC7469346

[8] Dahlawi HH, Al Obaidellah MM, Rashid NA, et al. Defining physician–nurse efforts toward collaboration as perceived by medical students. Healthcare (Basel). 2023;11(13):1919. doi: 10.3390/healthcare11131919 37444753 PMC10341369

[9] Lee S, Ryu Y, Kim J. The impacts of assertiveness on attitudes toward nurse-physician collaboration in nursing students. J Korean Acad Soc Nurs Educ. 2018;24(4):326–336. doi: 10.5977/jkasne.2018.24.4.326

[10] Baker L, Egan-Lee E, Martimianakis MA, et al. Relationships of power: implications for interprofessional education. J Interprof Care. 2011;25(2):98–104. doi: 10.3109/13561820.2010.505350 20846045

[11] Herath C, Zhou Y, Gan Y, et al. A comparative study of interprofessional education in global health care: a systematic review. Medicine [Baltimore]. 2017;96(38):e7336. doi: 10.1097/MD.0000000000007336 28930816 PMC5617683

[12] Lepage-Farrell A, Pinard AM, Richard A. Successful implementation of interprofessional education: a pedagogical design perspective. MedEdpublish (2016). 2024;14:55. doi: 10.12688/mep.20331.1 39416261 PMC11480705

[13] Kempner S, Brackmann M, Kobernik E, et al. The decline in attitudes toward physician-nurse collaboration from medical school to residency. J Interprof Care. 2020;34(3):373–379. doi: 10.1080/13561820.2019.1681947 31752567

[14] House S, Havens D. Nurses’ and physicians’ perceptions of nurse-physician collaboration: a systematic review. J Nurs Adm. 2017;47(3):165–171. doi: 10.1097/NNA.0000000000000460 28157818

[15] Collette A, Wann K, Nevin M, et al. An exploration of nurse-physician perceptions of collaborative behaviour. J Interprof Care. 2017;31(4):470–478. doi: 10.1080/13561820.2017.1301411 28394664

[16] Wang Y, Liu YF, Li H, et al. Attitudes toward physician-nurse collaboration in pediatric workers and undergraduate medical/nursing students. Behav Neurol. 2015;2015:1–6. doi: 10.1155/2015/846498 PMC4529947 26273131

[17] Sollami A, Caricati L, Mancini T. Ambivalent stereotypes of nurses and physicians: impact on students’ attitude toward interprofessional education. Acta Biomed. 2015;86(Suppl 1):19–28. 25835762

[18] Valenziano KB, Glod SA, Jia S, et al. An interprofessional curriculum to advance relational coordination and professionalism in early-career practitioners. MedEdPORTAL. 2018;14:10697. doi: 10.15766/mep_2374-8265.10697 30800897 PMC6342369

[19] Rudland J, Mires G. Characteristics of doctors and nurses as perceived by students entering medical school: implications for shared teaching. Med Educ. 2005;39(5):448–455. doi: 10.1111/j.1365-2929.2005.02108.x 15842678

[20] Helmich E, Derksen E, Prevoo M, et al. Medical students’ professional identity development in an early nursing attachment. Med Educ. 2010;44(7):674–682. doi: 10.1111/j.1365-2923.2010.03710.x 20636586

[21] Liaw SY, Siau C, Zhou WT, et al. Interprofessional simulation-based education program: a promising approach for changing stereotypes and improving attitudes toward nurse-physician collaboration. Appl Nurs Res. 2014;27(4):258–260. doi: 10.1016/j.apnr.2014.03.005 24849067

[22] Liaw SY, Wu LT, Lopez V, et al. Development and psychometric testing of an instrument to compare career choice influences and perceptions of nursing among healthcare students. BMC Med Educ. 2017;17(1):72. doi: 10.1186/s12909-017-0910-7 28449685 PMC5408422

[23] Zanotti R, Sartor G, Canova C. Effectiveness of interprofessional education by on-field training for medical students, with a pre-post design. BMC Med Educ. 2015;15(1):121. doi: 10.1186/s12909-015-0409-z 26220412 PMC4518727

[24] Thomas CD, DiSabatino L, Rojas F. The effects of education and clinical specialization on nurses’ status affirmation by physicians: a quantitative analysis. J Interprof Care. 2019;33(2):252–263. doi: 10.1080/13561820.2018.1544549 30444154

[25] Zakerimoghadam M, Ghiyasvandian S, Kazemnejad Leili A. Nurse-physician collaboration: the attitudes of baccalaureate nursing students at Tehran university of medical sciences. Iran Red Crescent Med J. 2015;17(4):e23247. doi: 10.5812/ircmj.17(4)2015.23247 26023338 PMC4443301

[26] Hansson A, Arvemo T, Marklund B, et al. Working together–primary care doctors’ and nurses’ attitudes to collaboration. Scand J Public Health. 2010;38(1):78–85. doi: 10.1177/1403494809347405 19762367

[27] Bales RF. Interaction process analysis: a method for the study of small groups. Cambridge: Addison Wesley Press; 1951.

[28] Bales RF. A set of categories for the analysis: a method for the study of small group interaction. Am Sociological Revoiew. 1950;15(2):257. doi: 10.2307/2086790

[29] Harris M, Greaves F, Gunn L, et al. Multidisciplinary group performance – measuring integration intensity in the context of the north west London integrated care pilot. Int J Integr Care. 2013;13(1):e001. doi: 10.5334/ijic.996 23687473 PMC3653286

[30] Campbell JD, Mauksch HO, Neikirk HJ, et al. Collaborative practice and provider styles of delivering health care. Soc Sci Med. 1990;30(12):1359–1365. doi: 10.1016/0277-9536(90)90316-k 2367881

[31] Peräkylä A. Two traditions of interaction research. Br J Soc Psychol. 2004;43(Pt 1):1–20. doi: 10.1348/014466604322915953 15035695

[32] Seelandt JC, Boos M, Kolbe M, et al. How to enrich team research in healthcare by considering five theoretical perspectives. Front Psychol. 2023;14:1232331. doi: 10.3389/fpsyg.2023.1232331 37637888 PMC10448055

[33] O’Brien BC, Harris IB, Beckman TJ, et al. Standards for reporting qualitative research: a synthesis of recommendations. Acad Med. 2014;89(9):1245–1251. doi: 10.1097/ACM.0000000000000388 24979285

[34] Braun V, Clarke V. Using thematic analysis in psychology. Qual Res Psychol. 2006;3(2):77–101. doi: 10.1191/1478088706qp063oa

[35] MacPhail C, Khoza N, Abler L, et al. Process guidelines for establishing intercoder reliability in qualitative studies. Qual Res. 2016;16(2):198–212. doi: 10.1177/1468794115577012

[36] Maher C, Hadfield M, Hutchings M, et al. Ensuring rigor in qualitative data analysis: a design research approach to coding combining NVivo with traditional material methods. Int J Qualitative Methods. 2018;17(1). doi: 10.1177/1609406918786362

[37] Asadi M, Ahmadi F, Mohammadi E, et al. Unsafe doctor-nurse interactions in the process of implementing medical orders: a qualitative study. Nurs Open. 2023;10(10):6808–6816. doi: 10.1002/nop2.1927 37353880 PMC10495711

[38] Klein A, Taieb O, Xavier S, et al. The benefits of mindfulness-based interventions on burnout among health professionals: a systematic review. Explore (NY). 2020;16(1):35–43. doi: 10.1016/j.explore.2019.09.002 31727578

[39] Berduzco-Torres N, Choquenaira-Callañaupa B, Medina P, et al. Factors related to the differential development of inter-professional collaboration abilities in medicine and nursing students. Front Psychol. 2020;11:432. doi: 10.3389/fpsyg.2020.00432 32292364 PMC7135885

[40] Tuirán-Gutiérrez GJ, San-Martín M, Delgado-Bolton R, et al. Improvement of inter-professional collaborative work abilities in Mexican medical and nursing students: a longitudinal study. Front Psychol. 2019;10:5. doi: 10.3389/fpsyg.2019.00005 30697172 PMC6340986

[41] Fox L, Onders R, Hermansen-Kobulnicky CJ, et al. Teaching interprofessional teamwork skills to health professional students: a scoping review. J Interprof Care. 2018;32(2):127–135. doi: 10.1080/13561820.2017.1399868 29172791

[42] Rosen MA, DiazGranados D, Dietz AS, et al. Teamwork in healthcare: key discoveries enabling safer, high-quality care. Am Psychol. 2018;73(4):433–450. doi: 10.1037/amp0000298 29792459 PMC6361117

[43] Nembhard I, Edmondson A. Making it safe: the effects of leader inclusiveness and professional status on psychological safety and improvement efforts in health care teams. J Organ Behav. 2006;27(7):941–966. doi: 10.1002/job.413

[44] Hansson A, Foldevi M, Mattsson B. Medical students’ attitudes toward collaboration between doctors and nurses – a comparison between two Swedish universities. J Interprof Care. 2010;24(3):242–250. doi: 10.3109/13561820903163439 19995272

